# Supplementary material for: Functional regulation of an ancestral RAG transposon ProtoRAG by a trans-acting factor YY1 in lancelet
Source: Nat Commun. 2020 Sep 9;11:4515. doi: 10.1038/s41467-020-18261-7 (PMC7481187; doi:10.1038/s41467-020-18261-7)
Supplement: Supplementary file 1 — Supplementary Information [file 41467_2020_18261_MOESM1_ESM.pdf]

Supplementary Information

Functional regulation of an ancestral RAG transposon *ProtoRAG* by a *trans*-acting factor YY1 in lancelet

Liu *et al*.

This SI file includes:

Supplementary Figures 1 to 7

Supplementary Table 1

Supplementary References

Other Supplementary Materials for this manuscript include the following:

Supplementary Data 1 (separate file). *Trans* factors on binding to core *cis* elements of *ProtoRAG* TIRs by JASPAR prediction.

Supplementary Figures

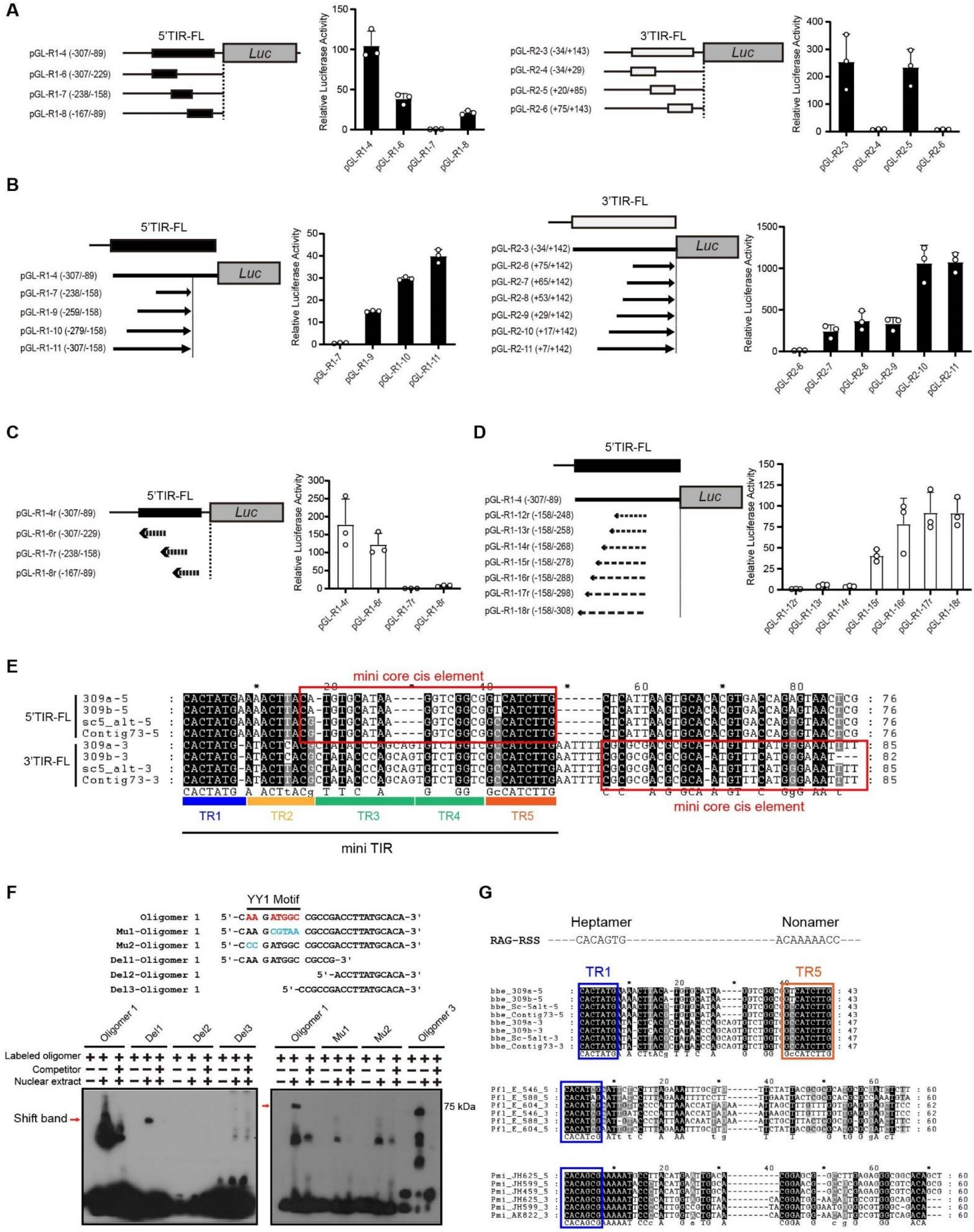

Supplementary Figure 1. Characterization of core *cis*-acting elements on *ProtoRAG* TIRs.

(A) The -307 to -229-bp sequence upstream of *bbRAG1L* TSS and the +20 to +85-bp sequence downstream of *bbRAG2L* TSS are regarded as efficient elements for the transcription of *bbRAG1L* and *bbRAG2L*. Results were obtained by luciferase reporter assays by transfecting the indicated TIR truncated plasmids into 293T cells.

(B) The -307 to -238-bp sequence upstream of *bbRAG1L* TSS and the +17 to +65-bp sequence downstream of *bbRAG2L* TSS are regarded as core elements for the transcription of *bbRAG1L* and *bbRAG2L*. Results were obtained by luciferase reporter assays by transfecting indicated TIR truncated plasmids into 293T cells.

(C) (D) Luciferase reporter assays using inverted TIR truncated plasmids indicated that the -298 to -268-bp sequence upstream of *bbRAG1L* TSS (+1) might be the mini core elements for the transcriptional activities of *bbRAG1L*.

(E) The mini core *cis* elements, mini-TIR and conserved TR1 and TR5 elements on 5'TIR-FL and 3'TIR-FL were as indicated.

(F) Representative EMSA showing that 293T cell nuclear extracts could not bind to the YY1 binding motif deletion or other mutated probes. EMSA was conducted by incubating 293T cell nuclear extracts with biotin-labeled probes in the absence or presence of excess competitor probe DNA. The results are from one representative experiment of two independent duplications with similar observations. Red, crucial sites for YY1 binding; blue, mutated nucleotides.

(G) TR1 of *ProtoRAG* is relatively conserved, while the 9-bp TR5 element of *ProtoRAG* is species specific. Sequences used for alignment are from publicly reported data[1, 2]. bbe, *B. belcheri*; Pfl, *P. flava*; Pmi, *P. minata*. Conserved nucleotide sequences are indicated in darker gray. For (A)-(D), the values are the means ±s.d., with n = 3 biologically independent experiments. For (A)-(D) and (F), source data are provided as Source Data file.

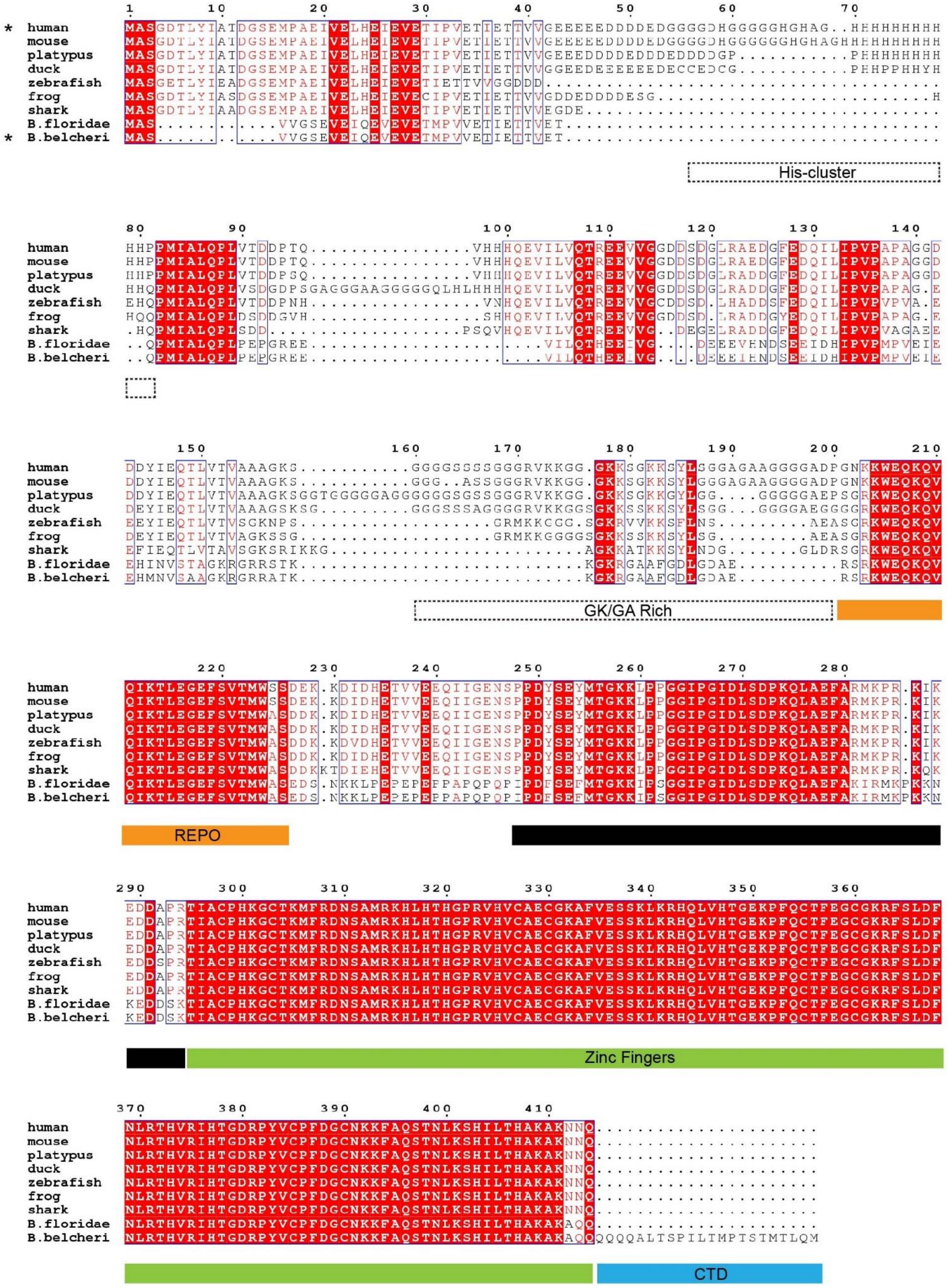
human
mouse
platypus
duck
zebrafish
frog
shark
B.floridae
B.belcheri
His-cluster
GK/GA Rich
REPO
Zinc Fingers
CTD

Supplementary Figure 2. Alignment of YY1-like proteins from representative species showed conservative domains in bbYY1.

The dashed box indicates the domains that exist in humans but are lacking in lancelet. Conservative amino acid sequences are colored in red. The accession numbers for YY1-like proteins include NP_003394.1 (human), NP_033563.2 (mouse), XP_028916341.1 (platypus), XP_027313566.1 (duck), NP_997782.1 (zebrafish), NP_001087404.1 (frog), XP_007886520.1 (shark), XP_002610706.1 (*Branchiostoma floridae*) and MF966513-MF966514 (*Branchiostoma belcheri*).

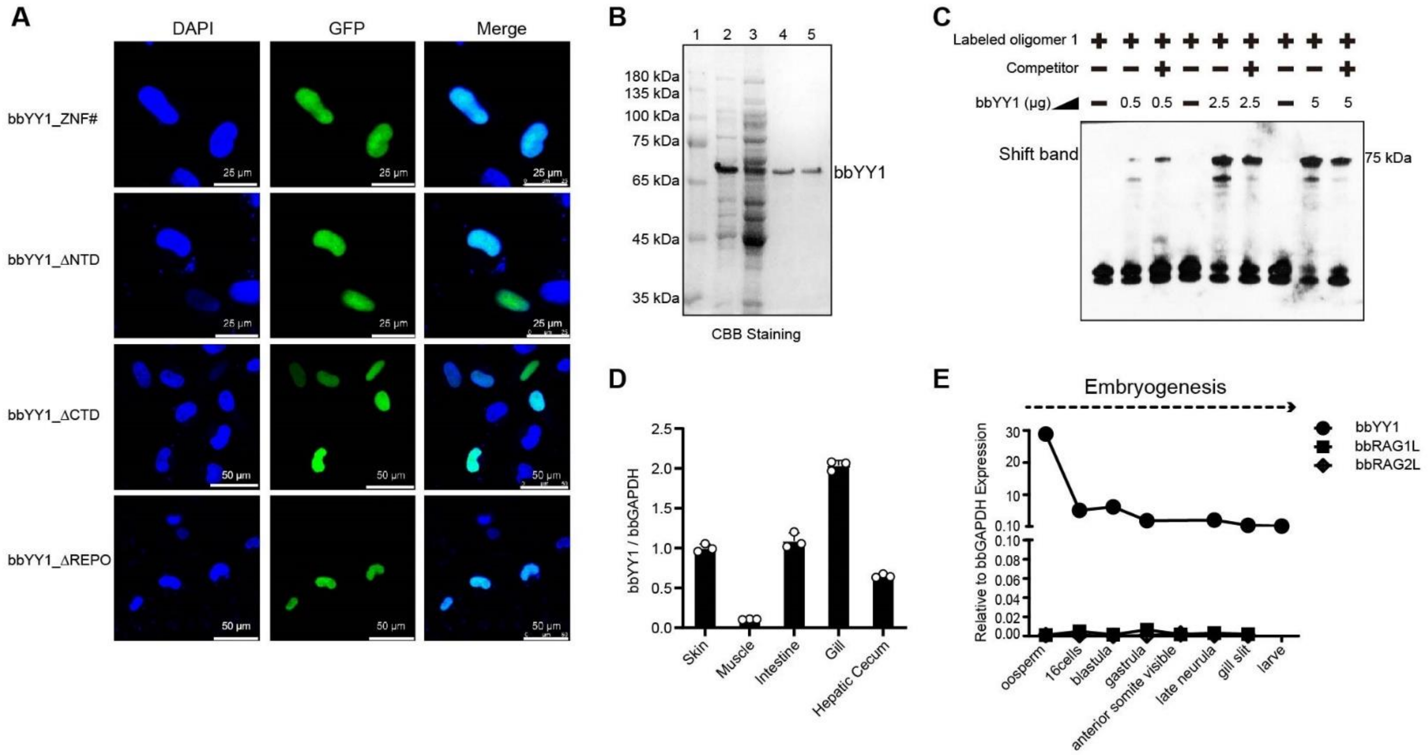

Supplementary Figure 3. Cellular and biochemical characterization of bbYY1.

(A) Laser confocal images suggested bbYY1 truncates are localized to the nucleus. Images display the GFP-tagged bbYY1 truncations in HeLa cells. These displayed images are from two independent repeated experiments with similar results.

(B) Prokaryotic expression and purification of recombinant bbYY1. Lane 1, protein marker; lane 2, supernatant from IPTG-induced bacteria after sonication; lane 3, flow through; lanes 4-5, elution with 160 mM and 250 mM imidazole, respectively.

(C) Representative EMSA gel indicating the binding between the bbYY1 and YY1-binding motif containing probe. (B) and (C) are one representative from three independent experiments.

(D) qPCR analysis of the expression of bbYY1 relative to bbGAPDH showing the abundance of *bbYY1* in adult lancelet tissues. The values are the means ± s.d., with n = 3 biologically independent experiments.

(E) Comparison of *bbYY1* and *bbRAG1L/bbRAG2L* mRNA expression levels during lancelet embryonic development stages. *BbRAG1L* and *bbRAG2L* mRNA expression data were obtained from a previously published article[1], and *bbYY1* mRNA expression data were obtained from the online lancelet genome database[3] [http://genome.bucm.edu.cn/lancelet]. For (B)-(E), source data are provided as Source Data file.

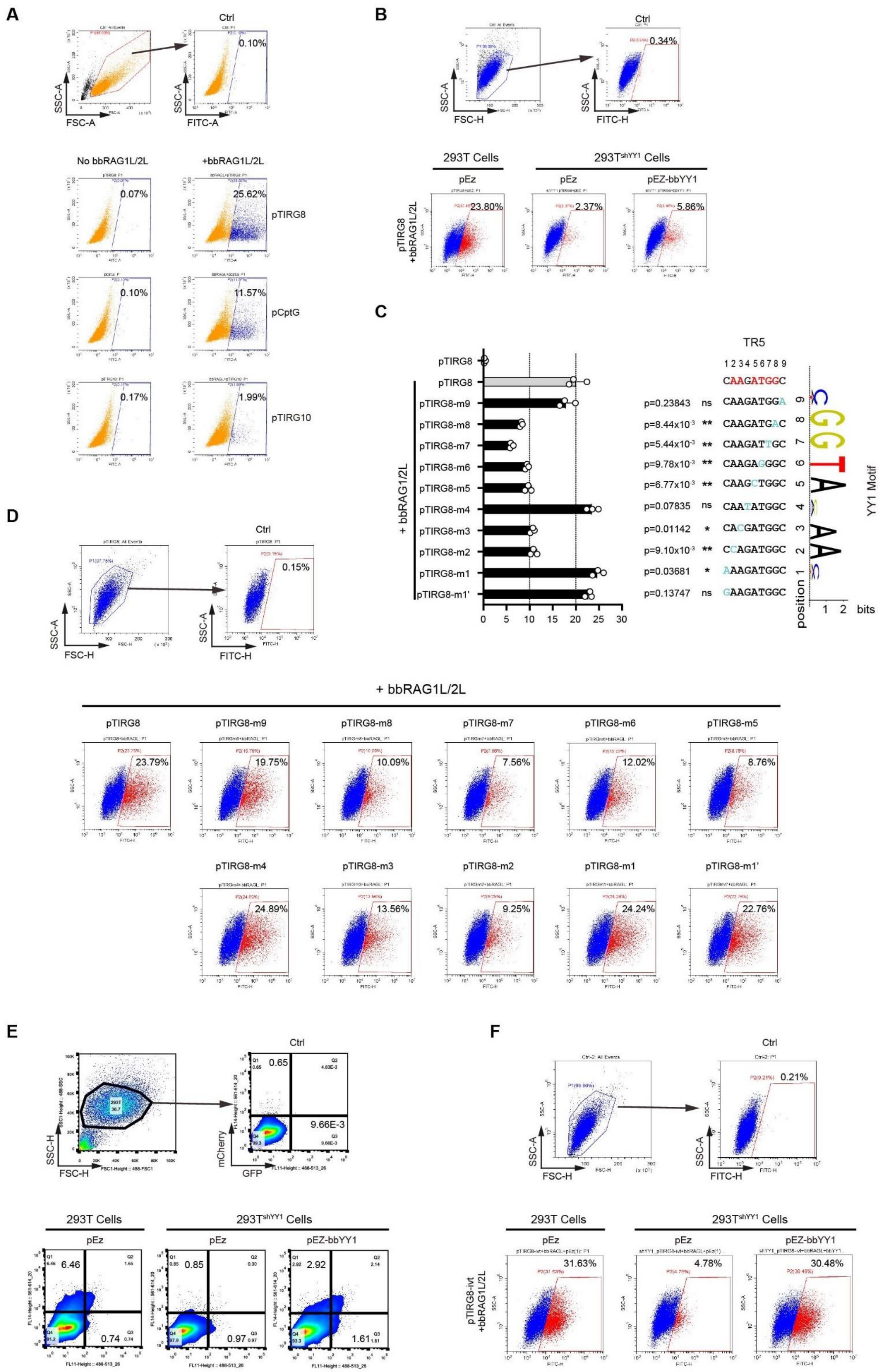

A
Ctrl
0.10%
SSC-A
FSC-A
FITC-A
No bbRAG1L/2L
+bbRAG1L/2L
0.07%
25.62%
pTIRG8
0.10%
11.57%
pCptG
0.17%
1.99%
pTIRG10
B
Ctrl
0.34%
SSC-A
FSC-H
FITC-H
293T Cells
293T^{shYY1} Cells
pEz
pEz
pEZ-bbYY1
pTIRG8 +bbRAG1L/2L
23.80%
2.37%
5.86%
C
TR5
1 2 3 4 5 6 7 8 9
pTIRG8
pTIRG8
pTIRG8-m9
pTIRG8-m8
pTIRG8-m7
pTIRG8-m6
pTIRG8-m5
pTIRG8-m4
pTIRG8-m3
pTIRG8-m2
pTIRG8-m1
pTIRG8-m1'
+bbRAG1/2L
CAAGATGGC
p=0.23843 ns CAAGATGGA
p=8.44x10-3 ** CAAGATGAC
p=5.44x10-3 ** CAAGATTGC
p=9.78x10-3 ** CAAGAGGGC
p=6.77x10-3 ** CAAGCTGGC
p=0.07835 ns CAATATGGC
p=0.01142 * CACGATGGC
p=9.10x10-3 ** CCAGATGGC
p=0.03681 * AAAGATGGC
p=0.13747 ns GAAGATGGC
0 5 10 15 20 25 30
position
1 2 bits
YY1 Motif
D
Ctrl
0.15%
SSC-A
FSC-H
FITC-H
+ bbRAG1L/2L
pTIRG8
23.79%
pTIRG8-m9
19.75%
pTIRG8-m8
10.09%
pTIRG8-m7
7.56%
pTIRG8-m6
12.02%
pTIRG8-m5
8.76%
pTIRG8-m4
24.89%
pTIRG8-m3
13.56%
pTIRG8-m2
9.25%
pTIRG8-m1
24.24%
pTIRG8-m1'
22.76%
E
Ctrl
0.65
9.66E-3
SSC-H
FSC-H
mCherry
GFP
293T Cells
293T^{shYY1} Cells
pEz
pEz
pEZ-bbYY1
6.46
0.74
0.85
0.97
2.92
1.61
F
Ctrl
0.21%
SSC-A
FSC-H
FITC-H
293T Cells
293T^{shYY1} Cells
pEz
pEz
pEZ-bbYY1
pTIRG8-ivt +bbRAG1L/2L
31.63%
4.78%
30.48%

Supplementary Figure 4. FACS analysis of TIR-dependent recombination and TTJ formation.

(A) The TR5 element is important for efficient TIR-dependent recombination mediated by the bbRAG1L/2L complex. Upper, gating strategy used to sort GFP-positive cells. Lower, representative flow cytometry statistics of GFP-positive cells after transfecting the indicated substrates (illustrated in Fig. 3d) into 293T cells.

(B) BbYY1 rescues the reduced recombination efficiency mediated by bbRAG1L/2L. Upper, gating strategy used to sort GFP-positive cells. Lower, representative flow cytometry statistics of GFP-positive cells after transfecting the indicated substrates. Related to Fig. 3e.

(C) Recombination efficiencies of TIR substrates correspond to conservation of nucleotides on the YY1 binding motif based on the flow cytometry analysis. Left, Histogram statistics of GFP-positive cells after transfecting bbRAGL with different TR5 element mutated substrates. Right, Illustration of different TR5 mutants. The red-colored nucleotides are the more highly conserved nucleotides in the YY1 binding motif with a higher position score. The light blue-colored nucleotides are mutation sites in the YY1 binding motif. The statistical values are the means ± s.d., with n = 3 biologically independent experiments. A two-tailed, unpaired Student's *t*-test was used for comparisons between the pTIRG8 and mutant group. *P<0.05; **P<0.01; ***P<0.001; ****P<0.0001. ns, not significant.

(D) Upper, gating strategy used to sort GFP-positive cells. Lower, representative flow cytometry statistics of GFP-positive cells after transfecting YY1 binding motif mutated substrates and bbRAG1L/2L expression vectors. Related to (C).

(E) Upper, gating strategy used for mCherry- and GFP-positive cells. Lower, representative flow cytometry results of mCherry- and GFP-positive cells after TIR-dependent recombination with transfection of indicated plasmids into 293T or $239T^{shYY1}$ cells. Related to Fig. 4e, f.

(F) Upper, gating strategy used to sort GFP-positive cells. Lower, representative flow cytometry statistics of GFP-positive cells after transfecting pTIRG8-ivt with the indicated protein expression vectors into 293T or $293T^{shYY1}$ cells. Related to Fig. 5a, b. Source data of (C) are provided as Source Data file.

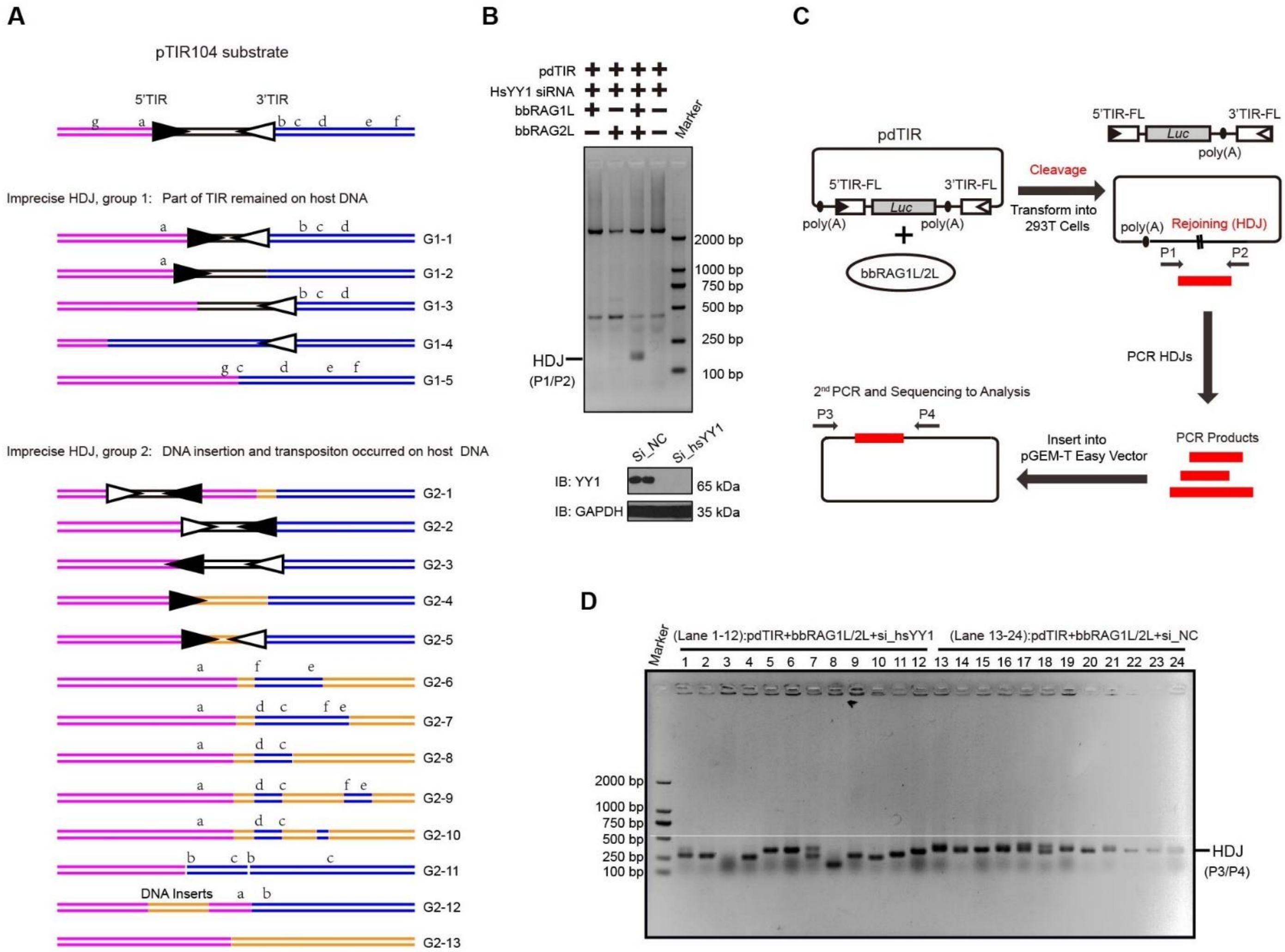

Supplementary Figure 5. YY1 plays roles in the formation of precise host DNA rejoining.

(A) Schematic diagram of different kinds of imprecise HDJs in our sequencing data. The orange lines indicate DNA inserts.

(B) Upper, PCR detection of HDJs after transfection of the indicated bbRAGL expression constructs and pdTIR together with hsYY1 siRNA into 293T cells. Lower, western blotting confirming the YY1 knockdown efficiency by hsYY1 siRNA. The results are one representative from three independent experiments.

(C) Flow chart of the PCR assay to detect HDJs in TIR-dependent recombination mediated by bbRAG1L/2L. P1/P2 and P3/P4 are PCR primers listed in Supplementary Table 1.

(D) Gel electrophoresis showed polymorphic HDJ product amplification by primers P3/P4 when hsYY1 was knocked down in 293T cells. The experiments were repeated three times independently with similar results. For (B) and (D), source data are provided as Source Data file.

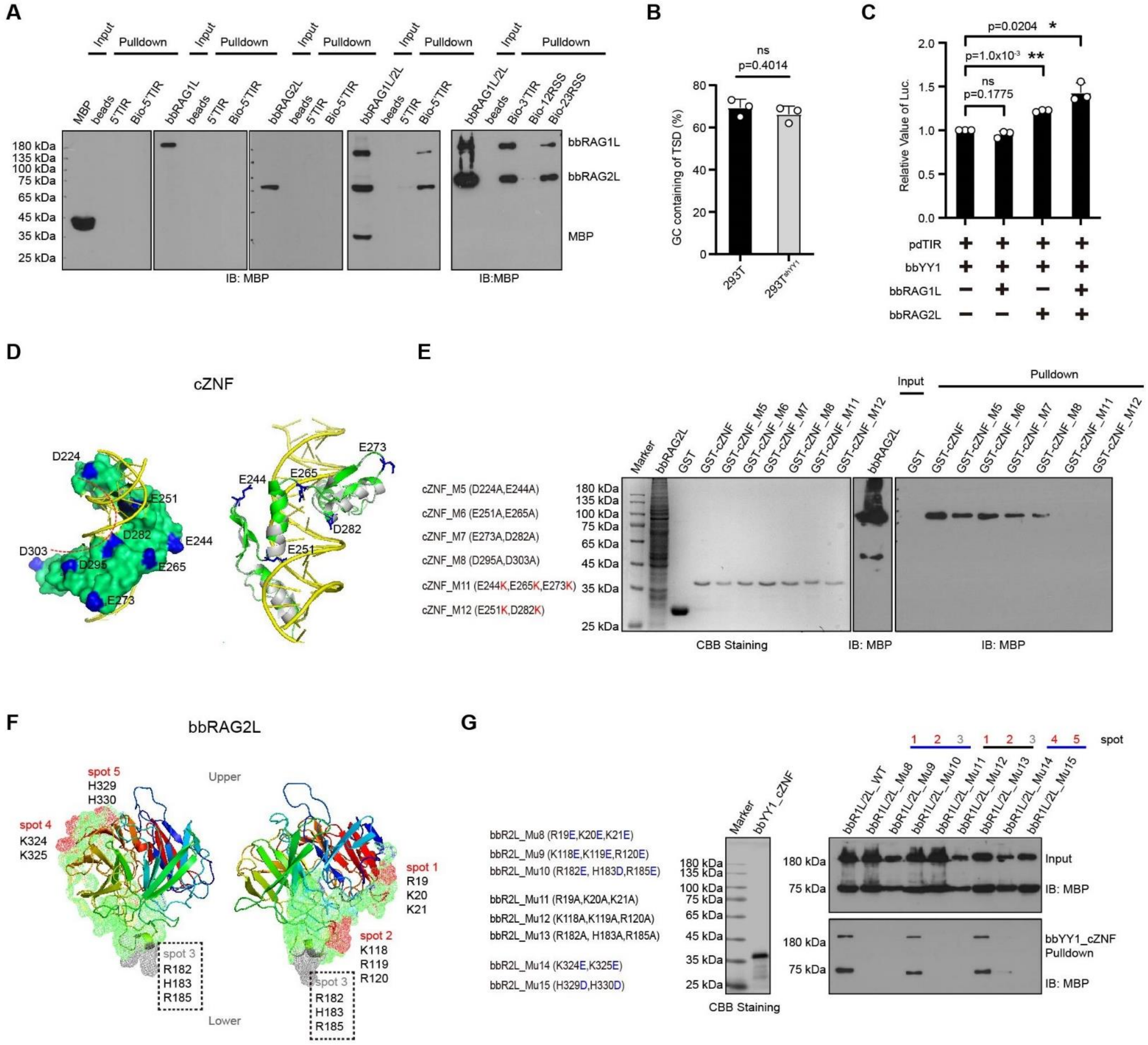

Supplementary Figure 6. Analysis of the interaction among bbRAG1L/2L, bbYY1 and TIR DNA.

(A) The DNA pulldown assay showed that bbRAG1L/2L could bind to 5' TIR, 3' TIR and 23-RSS. The apo bbRAG1L or bbRAG2L could not bind to the 5'TIR.

(B) GC containing TSD from intermolecular transposition products occurred in 293T and 293T^{shYY1} cells.

(C) The luciferase reporter assay suggested that bbRAG2L or bbRAG1L/2L attenuated bbYY1-mediated luciferase transcriptional inhibition, indicating a potential interaction between bbYY1 and bbRAG1L/2L *in vivo*. The luciferase reporter assays were performed by transfecting pdTIR and bbYY1 together with bbRAGL expression vectors into 293T cells.

(D) The surface and cartoon structure of the core zinc finger domain of bbYY1 (cZNF). The negatively charged residues aspartic and glutamic acid (abbreviated as "D" and "E", respectively) are colored in blue.

The cZNF of bbYY1 were modeled from zinc fingers of hsYY1[4] (green and gray colored; PDB: 1UBD [http://doi.org/10.2210/pdb1UBD/pdb]). The binding DNA of cZNF is colored in yellow.

(E) GST-pulldown showed that mutation of negatively charged residues on coils (between α-helixes and β-sheets) of the core zinc finger (cZNF) destroyed the interaction between cZNF and bbRAG2L.

(F) Cartoon-mesh structure of bbRAG2L. Spot 1 and spot 2 are located at the flank bottom of bbRAG2L. Spot 4 and spot 5 are on the edge of bbRAG2L. Spot 3 is located at a highly flexible loop area in the bbRAG2L crystal structure model[5] (PDB:6B40, [http://doi.org/10.2210/pdb6B40/pdb]), where it is hardly to determine its structural state.

(G) GST-pulldown showed that mutations of positively charged spots at the edge or bottom of bbRAG2L destroyed the interaction between bbRAG2L and cZNF. For (A), (E) and (G), the results are representative of three independent experiments. For (B) and (C), the values are the means ± s.d., with n = 3 biologically independent experiments. A two-tailed, unpaired Student's *t*-test was used for the comparisons. *P<0.05; **P<0.01; ***P<0.001; ****P<0.0001. ns, not significant. For (A)-(C), (E) and (G), source data are provided as Source Data file.

A

Protein Name	Protein ID & Domain Architecture *(H.sapiens)*	Protein ID & Domain Architecture *(B.belcheri)*
CREB	AAQ24858.1	Bb_213500F
ATF2 iso 1	NP_001871.2	Bb_199390R
ATF2 iso 2	NP_001243020.1	
ATF2 iso 3	NP_001243021.1	
ATF2 iso 4	NP_001243022.1	
ATF2 iso 5	NP_001243023.1	
ATF4	NP_001666.2	Bb_183020F
ATF6	EAW90694.1	Bb_012790R
ATF6B	AAH77075.1	

B

Protein Category	Protein Name	Protein ID & Domain Architecture *(H.sapiens)*	Protein ID & Domain Architecture *(B.belcheri)*
PRC1	CBX2	NP_005180.1	074100F
	CBX4	NP_003646.2	
	CBX6	NP_055107.3	
	CBX7	NP_783640.1	
	CBX8	NP_065700.1	
	PH1	gb:AAI57851.1	087130F
	PH2	gb:AAI10864.2	
	PH3	emb:CAC86587.2	
	PCGF1	NP_116062.2	126940F
	PCGF5	NP_001244030.1	
	PCGF2	NP_009075.1	233040R
	PCGF4	NP_005171.4	
	PCGF3	NP_001304765.1	304250F
	PCGF6	gb：AAH10235.1	136680F
	RING1A	NP_002922.2	013490R
	RING1B	NP_009143.1	
PRC2	EZH1	NP_001982.2	014440F
	EZH2	NP_004447.2	
	SUZ12	NP_056170.2	302410F
	EED	NP_003788.2	103590F
	RBBP7	NP_005601.1	059690R
	RBBP4	NP_001185648.1	

Supplementary Figure 7. Homologues of YY1 co-repressors in *H. sapiens* and *B. belcheri*.

(A) The homologues of cAMP responsive element binding protein/activating transcription factor (CREB/ATF) in *H. sapiens* and *B. belcheri*.

(B) The homologues of polycomb repressive complex (PRC) in *H. sapiens* and *B. belcheri*.

Supplementary Tables

Name	Sequence (5'-3')
Oligomer 1	CAAGATGGCCGCCGACCTTATGCACA
Oligomer 2	TGAAACATTGCGCGTCGC
Oligomer 3	ATTGCGCGTCGCGCGAAAATTCAAGATGGCGACCAGACACTGC
Mu1-oligmer1	CAAGCGTTACGCCGACCTTATGCACA
Mu2-oligmer1	CCCGATGGCCGCCGACCTTATGCACA
Del1-oligmer1	CAAGATGGCCGCCG
Del2-oligmer1	ACCTTATGCACA
Del3-oligmer1	CCGCCGACCTTATGCACA
shRNA hsYY1#1-F	CCGGGCCTCTCCTTTGTATATTATTCTCGAGAATAATATACAAAGGAGAGGCTTTTTG
shRNA hsYY1#1-R	AATTCAAAAAGCCTCTCCTTTGTATATTATTCTCGAGAATAATATACAAAGGAGAGGC
shRNA hsYY1#2-F	CCGGCTGGCAGAATTTGCTAGAATGCTCGAGCATTCTAGCAAATTCTGCCAGTTTTTG
shRNA hsYY1#2-R	AATTCAAAAACTGGCAGAATTTGCTAGAATGCTCGAGCATTCTAGCAAATTCTGCCAG
shRNA hsYY1#3-F	CCGGGGAGCAGAAGCAGGTGCAGATCTCGAGATCTGCACCTGCTTCTGCTCCTTTTTG
shRNA hsYY1#3-R	AATTCAAAAAGGAGCAGAAGCAGGTGCAGATCTCGAGATCTGCACCTGCTTCTGCTCC
pGL$_3$-CMV-F	AAGGTACCATAGTAATCAATTACGGGGTCATTAGTTCATAGC
pGL$_3$-CMV-R	AAGAGCTCGATCTGACGGTTCACTAAACCAGCT
pGL$_3$-SV40-F	AAGGTACCTGTGTCAGTTAGGGTGTGGAAAGT
pGL$_3$-SV40-R	AAGAGCTCTAGCTCAGAGGCCGAGGCG
5'bbYY1-RGSP	TGCTGCTGTTGTTGCTGGGCCTTG
5'bbYY1-RNeGSP-1	GGCCCGTGTGTGTGCAGGTGC
3'bbYY1-FGSP	TCCACGTCTGCGCCGAATGTGGA
3'bbYY1-FNeGSP-1	CCCTACGTCTGCCCCTTCGACGGC
P1	CGGGAGGTACTTGGAGCGGCC
P2	TGCCGGCACCTGTCCTACGAG
P3	TAATACGACTCACTATAGGG
P4	ATTTAGGTGACACTATAGAA
P5	CGTGTGCATAAGGTCGGCG
P6	CGGTGACTCGGGAGACTAGT
P7	GCTATACCCAGCAGTGTCTGGTC
FM1-Linker	CCATACTAGTC
FM2-Linker	GCGGTGACTCGGGAGACTAGTATGG
pGL_5'RNGSP	TATGCAGTTGCTCTCCAGCGGTTCC
pGL_5'RGSP	TCATAGCTTCTGCCAACCGAACGGACA
pGL_5'RGSP-1	AATAACGCGCCCAACACCGGC
pTIR104-F	GCGCCCAATACGCAAACCGCCT
pTIR104-R	TTCCGGATGAGCATTCATCAGGCGGG
pJH_P2 Primer	GGGATATATCAACGGTGGTATATCCAGTG
RT_bbGAPDH_U	CAAGGCTGTAGGCAAGGTCAT
RT_bbGAPDH_L	CTTCTTCAGTCGGCAGGTCAG
RT_bbYY1-U	AGACAACTCTGCGATGCGGA
RT_bbYY1-L	TTCCCGCAACCCTCAAACGT

Supplementary Table 1. Primers and oligomers used in this study.

Primers named as oligomers were used in the EMSA. Primers named as shRNA hsYY1 were used for the construction of the pLKO.1-shYY1 vector. Primers named as bbYY1-GSP were used in the RACE PCR assays for the cloning of the bbYY1 gene. P1-P7 primers were used for the detection of HDJs or TIREs. FM-Linker primers were used for the preparation of the adaptor in the LM-PCR assays. Primers named as pGL_GSP were the specific primer for 5'RACE PCR for identification of TSSs of *bbRAG1L* or *bbRAG2L*. pTIR104 or pJH primers were the sequencing primers for the identification of cam^R/Kan^R -positive bacteria colonies. RT primers were used in qPCR assays for the quantification of the expression of the *bbYY1* gene.

Supplementary References

1. Huang S*, et al.* Discovery of an active RAG transposon illuminates the origins of V(D)J recombination. *Cell* **166**, 102-114 (2016).

2. Morales Poole JR, Huang SF, Xu A, Bayet J, Pontarotti P. The RAG transposon is active through the deuterostome evolution and domesticated in jawed vertebrates. *Immunogenetics* **69**, 391-400 (2017).

3. You L, *et al*. LanceletDB: an integrated genome database for lancelet, comparing domain types and combination in orthologues among lancelet and other species. *Database(Oxford)*. doi:10.1093/database/baz056 (2019).

4. Houbaviy HB, Usheva A, Shenk T, Burley SK. Cocrystal structure of YY1 bound to the adeno-associated virus P5 initiator. *Proc. Natl. Acad. Sci. U S A* **93**, 13577-13582 (1996).

5. Zhang Y, et al. Transposon molecular domestication and the evolution of the RAG recombinase. *Nature* **569**, 79-84 (2019).
