# Supplementary material for: Functional regulation of an ancestral RAG transposon ProtoRAG by a trans-acting factor YY1 in lancelet
Source: Nat Commun. 2020 Sep 9;11:4515. doi: 10.1038/s41467-020-18261-7 (PMC7481187; doi:10.1038/s41467-020-18261-7)
Supplement: Supplementary file 3 — Reporting Summary [file 41467_2020_18261_MOESM3_ESM.pdf]

nature research

Corresponding author(s): Anlong Xu

Last updated by author(s): Aug 29, 2020

Reporting Summary

Nature Research wishes to improve the reproducibility of the work that we publish. This form provides structure for consistency and transparency in reporting. For further information on Nature Research policies, see Authors & Referees and the Editorial Policy Checklist .

Statistics

For all statistical analyses, confirm that the following items are present in the figure legend, table legend, main text, or Methods section.

n/a	Confirmed	
☐	☒	The exact sample size (n) for each experimental group/condition, given as a discrete number and unit of measurement
☐	☒	A statement on whether measurements were taken from distinct samples or whether the same sample was measured repeatedly
☐	☒	The statistical test(s) used AND whether they are one- or two-sided<br>*Only common tests should be described solely by name; describe more complex techniques in the Methods section.*
☒	☐	A description of all covariates tested
☐	☒	A description of any assumptions or corrections, such as tests of normality and adjustment for multiple comparisons
☐	☒	A full description of the statistical parameters including central tendency (e.g. means) or other basic estimates (e.g. regression coefficient) AND variation (e.g. standard deviation) or associated estimates of uncertainty (e.g. confidence intervals)
☐	☒	For null hypothesis testing, the test statistic (e.g. F, t, r) with confidence intervals, effect sizes, degrees of freedom and P value noted<br>*Give P values as exact values whenever suitable.*
☒	☐	For Bayesian analysis, information on the choice of priors and Markov chain Monte Carlo settings
☒	☐	For hierarchical and complex designs, identification of the appropriate level for tests and full reporting of outcomes
☒	☐	Estimates of effect sizes (e.g. Cohen's d, Pearson's r), indicating how they were calculated

Our web collection on statistics for biologists contains articles on many of the points above.

Software and code

Policy information about availability of computer code

Data collection	The luciferase reporter data were collected by using the Glomax 20/20 luminometer system (Promega). The flow cytometry data were collected by using CytoFLEX and MoFlo Astrios EQs instruments (Beckman). qPCR data were obtained from the Roche's LightCycler 480 real-time PCR system. The confocal immunofluorescence data were captured by using a LEICA TCS-SP5 microscope. Electrophoresis gels were imaged by using a Molecular Imager VersaDoc™ MP 4000 System (Bio-Rad) or a SynGene image system.
Data analysis	DNA sequencing data were analyzed by using APE v2.0.49.10 and SeqMan softwares 7.1.0 (44.1) (Gene Star). The alignment of DNA or protein sequences was performed using CLUSTALW 2.1, MEGA5.2 or T-Coffee server (ref.57) [http://www.tcoffee.org/]. The refinement or shading of sequence alignments was performed using the Genedoc 2.7, BioEdit software7.0.5.2 or the ESPript 3.0 server (ref.54) [http://espript.ibcp.fr/ESPript/cgi-bin/ESPript.cgi]. Homologous domain architectures predictions were performed on the SMART server (ref.56) [http://smart.embl-heidelberg.de/]. A homologous model of bbYY1 was predicted by the TASSER program (ref.59) [https://zhanglab.ccmb.med.umich.edu/I-TASSER/]. The bbRAG2L and bbYY1_cZNF were displayed by PyMOL software (TM) 1.7.4.5 Edu. Statistical analysis was performed using GraphPad Prism 8 or Microsoft Excel 2013. The flow cytometry data was analyzed by CytExpert 1.2 and FlowJo 10 software.

For manuscripts utilizing custom algorithms or software that are central to the research but not yet described in published literature, software must be made available to editors/reviewers. We strongly encourage code deposition in a community repository (e.g. GitHub). See the Nature Research guidelines for submitting code & software for further information.

Data

Policy information about availability of data

All manuscripts must include a data availability statement. This statement should provide the following information, where applicable:

- Accession codes, unique identifiers, or web links for publicly available datasets
- A list of figures that have associated raw data
- A description of any restrictions on data availability

The data that support the findings of this study are available within the article and Supplementary Information. The bbYY1 cDNA sequences have been deposited in the GenBank database under accession numbers MF966513[https://www.ncbi.nlm.nih.gov/nuccore/MF966513] and MF966514[https://www.ncbi.nlm.nih.gov/nuccore/MF966514]. Partial sequences of the BAC plasmid clone BAC73, which contains the complete coding sequences (CDS) of the bbRAG1L and bbRAG2L genes, are available in DDBJ/ENA/GenBank under accession number KJ748699 [https://www.ncbi.nlm.nih.gov/nuccore/KJ748699] with identifiers (ref. 6). The 3D model structures of the human YY1 zinc finger and bbRAGL which supported this study are available from the PDB database under the accession codes 1UBD [http://doi.org/10.2210/pdb1UBD/pdb] and 6B40 [http://doi.org/10.2210/pdb6B40/pdb] respectively, with the identifiers (ref. 7, ref. 58). The source data are provided with this paper. Further data are available from the corresponding author upon request.

Field-specific reporting

Please select the one below that is the best fit for your research. If you are not sure, read the appropriate sections before making your selection.

- [x] Life sciences
- [ ] Behavioural & social sciences
- [ ] Ecological, evolutionary & environmental sciences

For a reference copy of the document with all sections, see nature.com/documents/nr-reporting-summary-flat.pdf

Life sciences study design

All studies must disclose on these points even when the disclosure is negative.

Sample size	The sample sizes in our experiments were determined based on our experience and general standards in the field. This type of experience is necessary to generate a convincing and compelling result. The experiments, which were general quantification to different groups, were repeated three times independently (n=3), and each was performed using triplicate samples. Similar methods can be found in the ref. 6, ref. 8. For the blotting analysis, the representative results are from two or three independently performed experiments. A Similar method could be found in ref. 6, ref. 7. The number of independent experiments is indicated in each figure legend.
Data exclusions	No data were excluded from the analyses.
Replication	Experiments are reliably reproduced. Generally, experiments were performed at least three times, unless otherwise noted in the manuscript or figure legends.
Randomization	Randomization was not relevant to our study because the study did not involve the allocation of samples into experimental groups.
Blinding	No blinding was performed in this study because group allocation was not involved in our study. The researchers were not blinded during data collection because most of the measurements were performed using instruments, or were quantitative in nature (blots, gels or numbers of colonies on a plate), and these data types are not easy to subjectively interpret.

Reporting for specific materials, systems and methods

We require information from authors about some types of materials, experimental systems and methods used in many studies. Here, indicate whether each material, system or method listed is relevant to your study. If you are not sure if a list item applies to your research, read the appropriate section before selecting a response.

Materials & experimental systems

n/a	Involved in the study	
[ ]	[x]	Antibodies
[ ]	[x]	Eukaryotic cell lines
[x]	[ ]	Palaeontology
[ ]	[x]	Animals and other organisms
[x]	[ ]	Human research participants
[x]	[ ]	Clinical data

Methods

n/a	Involved in the study	
[x]	[ ]	ChIP-seq
[ ]	[x]	Flow cytometry
[x]	[ ]	MRI-based neuroimaging

Antibodies

Antibodies used	anti-Flag (1: 5,000; 66008-2-Ig, Proteintech)<br>anti-GAPDH (1:10,000; 60004-1-Ig, Proteintech)

	anti-GST (1: 5,000; 71097-3, Merck)<br>anti-His (1: 5,000; 70796-3, Merck)<br>anti-MBP (1: 5,000; 66003-1-Ig, Proteintech) or (1: 5,000; M 6295, Sigma).<br>anti-YY1 (1: 5,000; H414; sc-1703, Santa Cruz) or (1: 5,000; C20; sc-281, Santa Cruz)<br>goat anti-mouse IgG-HRP (1:10,000; HA1006, HuaBio)<br>goat anti-rabbit IgG-HRP (1:10,000; HA1001, HuaBio)
Validation	All antibodies used in our study were purchased from the indicated commercial manufacturers and validated according to their instructions. The specificities of the anti-YY1 and anti-GAPDH antibodies were verified as single intense bands of the expected molecular weights by western blotting (refer to Fig. 2e, f). The specificities of the anti-his, anti-flag, anti-GST, and anti-MBP were verified using his-tagged protein, flag-tagged protein, gst-tagged protein and MBP-tagged protein in western blotting. These antibodies can used in multiple mouse and human cell lines according their introductions indicated on the manufacturer's website.

Eukaryotic cell lines

Policy information about cell lines

Cell line source(s)	HEK293T and Hela cells used in this study were obtained from ATCC. 293TshYY1 cells were generated in this study.
Authentication	The 293T and Hela cell lines from ATCC were authenticated by short tandem repeat profiling. Based on the morphological features of these cells, microscopic inspection was also used to distinguish these two cell types. The 293TshYY1 cells were confirmed by western blotting (Fig 2f).
Mycoplasma contamination	Cell lines used in this study tested negative for mycoplasma.
Commonly misidentified lines (See ICLAC register)	No commonly misidentified cell lines were used in this study.

Animals and other organisms

Policy information about studies involving animals; ARRIVE guidelines recommended for reporting animal research

Laboratory animals	The study did not involve laboratory animals.
Wild animals	Adult Chinese amphioxus (~1 year old) B. belcheri were captured using dense nets from the sea area near Zhanjiang city, China. After capturing, the amphioxus were put into a sea water-containing tank and transported to the laboratory. During transportation, the sea water container was kept at 18-25°C. The captured amphioxus were cultured in a laboratory incubator under modeled wild conditions. For RNA extraction and bbYY1 gene cloning, adult amphioxus were randomly selected, quickly frozen and euthanized by liquid nitrogen.
Field-collected samples	No field-collected samples were used in this study.
Ethics oversight	All the animal experiments were approved by the Institutional Animal Care and Use Committee of Sun Yat-Sen University.

Note that full information on the approval of the study protocol must also be provided in the manuscript.

Flow Cytometry

Plots

Confirm that:

- [x] The axis labels state the marker and fluorochrome used (e.g. CD4-FITC).
- [x] The axis scales are clearly visible. Include numbers along axes only for bottom left plot of group (a 'group' is an analysis of identical markers).
- [x] All plots are contour plots with outliers or pseudocolor plots.
- [x] A numerical value for number of cells or percentage (with statistics) is provided.

Methodology

Sample preparation	HEK293T or 293TshYY1 cells were transfected with EGFP or mCherry expression plasmids, indicated bbRAGL and YY1 expression plasmids, then cultured for another 48hrs. These cells were digested with trypsin, gathered and washed, resuspended in cold PBS buffer for flow cytometry analysis.
Instrument	CytoFLEX and MoFlo Astrios EQs (Beckman)
Software	Flow Jo 10 and CytExpert 1.2 software were used for data analysis.

Cell population abundance	The purity of the sorted 293T or 293TshYY1 cells fractions was >95%, and it was determined by setting FSC/SSC gates on flow cytometry.
Gating strategy	The pTIRG and pEGFPN1 plasmid were transfected into 293T cells for gating the negative and positive cell populations respectively. GFP positive signals of the pTIRG transfecting cells that gated less than 1% were determined as a negative cell population. A representative plot and gating are provided within related flow cytometry figure.

☒ Tick this box to confirm that a figure exemplifying the gating strategy is provided in the Supplementary Information.
