# Supplementary material for: Functional regulation of an ancestral RAG transposon ProtoRAG by a trans-acting factor YY1 in lancelet
Source: Nat Commun. 2020 Sep 9;11:4515. doi: 10.1038/s41467-020-18261-7 (PMC7481187; doi:10.1038/s41467-020-18261-7)
Supplement: Supplementary file 4 — Description of Additional Supplementary Files [file 41467_2020_18261_MOESM4_ESM.pdf]

Description of Additional Supplementary Files

Supplementary Data 1 . *Trans* factors on binding to core *cis* elements of *ProtoRAG* TIRs by JASPAR prediction.
